# 1-Bromo-2,7-di-*tert*-butyl­pyrene

**DOI:** 10.1107/S1600536809054257

**Published:** 2009-12-19

**Authors:** Guang-Ming Xia, Zhi-Qiang Liu, Ping Lu, Guo-Xin Sun, Hong-Yu Chen

**Affiliations:** a School of Chemistry and Chemical Engineering, University of Jinan, Ji’nan 250022, People’s Republic of China; b State Key Laboratory of Crystal Materials, Shandong University, Jinan 250100, Shandong Province, People’s Republic of China; c School of Chemistry and Chemical Engineering, TaiShan Medical University, Tai’an 271016, People’s Republic of China

## Abstract

In the title mol­ecule, C_24_H_25_Br, one of two *tert*-butyl groups is rotationally disordered between two orientations in a 0.59 (3):0.41 (3) ratio. The crystal packing exhibits no π–π inter­actions; however, relatively short inter­molecular Br⋯Br contacts of 3.654 (1) Å are observed.

## Related literature

For the synthesis, see: Yamato *et al.* (1997 ▶). For a related structure, see: Hazell & Lomborg (1972 ▶).
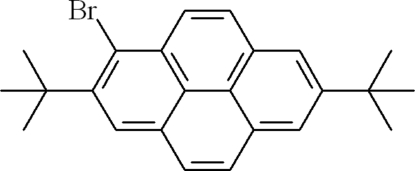


## Experimental

### 

#### Crystal data

C_24_H_25_Br
*M* *_r_* = 393.35
Orthorhombic, 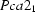

*a* = 21.4678 (4) Å
*b* = 14.5221 (2) Å
*c* = 6.2436 (1) Å
*V* = 1946.49 (5) Å^3^
*Z* = 4
Mo *K*α radiation
μ = 2.12 mm^−1^
*T* = 293 K
0.32 × 0.21 × 0.13 mm

#### Data collection

Bruker APEXII CCD area-detector diffractometer
Absorption correction: multi-scan (*SADABS*; Bruker, 2005 ▶) *T*_min_ = 0.643, *T*_max_ = 0.651
15786 measured reflections
4402 independent reflections
2741 reflections with *I* > 2σ(*I*)
*R*_int_ = 0.037

#### Refinement

*R*[*F* ^2^ > 2σ(*F* ^2^)] = 0.090
*wR*(*F* ^2^) = 0.281
*S* = 1.00
4402 reflections
263 parameters
67 restraints
H-atom parameters constrained
Δρ_max_ = 1.01 e Å^−3^
Δρ_min_ = −0.84 e Å^−3^
Absolute structure: Flack (1983 ▶), 1930 Friedel pairs
Flack parameter: 0.05 (3)

### 

Data collection: *APEX2* (Bruker, 2005 ▶); cell refinement: *SAINT* (Bruker, 2005 ▶); data reduction: *SAINT*; program(s) used to solve structure: *SIR97* (Altomare *et al.*, 1999 ▶); program(s) used to refine structure: *SHELXL97* (Sheldrick, 2008 ▶); molecular graphics: *SHELXTL* (Sheldrick, 2008 ▶); software used to prepare material for publication: *WinGX* (Farrugia, 1999 ▶).

## Supplementary Material

Crystal structure: contains datablocks global, I. DOI: 10.1107/S1600536809054257/cv2677sup1.cif

Structure factors: contains datablocks I. DOI: 10.1107/S1600536809054257/cv2677Isup2.hkl

Additional supplementary materials:  crystallographic information; 3D view; checkCIF report

## Figures and Tables

**Fig. 1.**
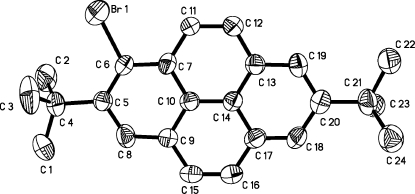
The molecular structure of the title compound showing the atomic numbering and 50% probability displacement ellipsoids. Only major parts of disordered atoms are shown. H atoms omitted for clarity.

## supplementary crystallographic information

### Comment

Pyrene and its derivatives are often used as fluorescent chromophores. Normally, the electrophilic substitution of pyrene occurred at positions 1, 3, 6 or 8 position, but not at other positions (2,4,5,7,9 and 10). However, the orientation in friedel-crafts *tert*-butylation of pyrene have been proved at positions 2 and 7. Yamato and co workers had reported that the bromination of 2,7-di-*tert*-butylpyrene with 1 mol equiv of bromine in carbon tetrachloride solution afford 1-bromo-2,7-di-*tert*-butylpyrene in high yield (Yamato *et al.*, 1997). However, no crystal data were given as a proof. Herein, we report the crystal structure of 1-bromo-2,7-di-*tert*-butylpyrene, (I), which support the conclusion of Yamato.

In (I) (Fig. 1), all bond lengths and angles are normal and comparable to those reported for close compound (Hazell *et al.*, 1972). One of two *tert*-butyl groups (attached to pyrene at position 7) is rotationally disordered between two orientations in a ratio 0.59 (3):0.41 (3). The crystal packing exhibits no π-π interactions, however, relatively short intermolecular Br···Br contacts of 3.654 (1) are observed.

### Experimental

The title compound was synthesized by the bromination of 2,7-di-*tert*-butylpyrene. To a solution of 2,7-di-*tert*-butylpyrene(314 mg, 1.0 mmol) in 30 ml CCl_4_, a solution of Br_2_ (200 mg, 1.1 mmol) in 10 ml CCl_4_ was added at 0°C. After the reaction mixture had been stirred for 1 h at room temperature, it was poured into water and the organic layer was extracted with CH_2_Cl_2_ and washed with solution of sodium thiosulfate and water, dried over MgSO_4_ and concentrated. The residue was purified by silica gel column chromatography with hexane as eluent t o afford a solid. Recrystallization from ethanol gave the 1-bromo-2,7-di-*tert*-butylpyrene(yield: 290 mg, 75%) as colorless prism crystals.

### Refinement

All H atoms were geometrically fixed and allowed to ride on their attached atoms, which C—H = 0.93 Å and *U*_iso_(H)= 1.2 *U*_eq_(C) for the H-atom bonded to thiophene ring, N—H= 0.86Å and *U*_iso_(H)= 1.2 *U*_eq_(C) and the other C—H = 0.93 Å and *U*_iso_(H)= 1.5 *U*_eq_(C). Tert-butyl group (attached to C20) is disordered between two orientations. Three methyl groups - C22, C23, C24 - were refined to a rigid model around the bond C20—C21 with methyl groups C22', C23' and C24', with the occupancies refined to 0.41 (3) and 0.59 (3), respectively.

### Crystal data

**Table:**

C_24_H_25_Br	*F*(000) = 816
*M**_r_* = 393.35	*D*_x_ = 1.342 Mg m^−^^3^
Orthorhombic, *P**c**a*2_1_	Mo *K*α radiation, λ = 0.71073 Å
Hall symbol: P 2c -2ac	Cell parameters from 3661 reflections
*a* = 21.4678 (4) Å	θ = 2.8–21.3°
*b* = 14.5221 (2) Å	µ = 2.12 mm^−^^1^
*c* = 6.2436 (1) Å	*T* = 293 K
*V* = 1946.49 (5) Å^3^	Prism, colourless
*Z* = 4	0.32 × 0.21 × 0.13 mm

### Data collection

**Table:**

Bruker APEXII CCD area-detector diffractometer	4402 independent reflections
Radiation source: fine-focus sealed tube	2741 reflections with *I* > 2σ(*I*)
graphite	*R*_int_ = 0.037
φ and ω scans	θ_max_ = 27.5°, θ_min_ = 1.7°
Absorption correction: multi-scan (*APEX2*; Bruker, 2005)	*h* = −25→27
*T*_min_ = 0.643, *T*_max_ = 0.651	*k* = −18→18
15786 measured reflections	*l* = −8→8

### Refinement

**Table:**

Refinement on *F*^2^	Secondary atom site location: difference Fourier map
Least-squares matrix: full	Hydrogen site location: inferred from neighbouring sites
*R*[*F*^2^ > 2σ(*F*^2^)] = 0.090	H-atom parameters constrained
*wR*(*F*^2^) = 0.281	*w* = 1/[σ^2^(*F*_o_^2^) + (0.1998*P*)^2^] where *P* = (*F*_o_^2^ + 2*F*_c_^2^)/3
*S* = 1.00	(Δ/σ)_max_ < 0.001
4402 reflections	Δρ_max_ = 1.01 e Å^−^^3^
263 parameters	Δρ_min_ = −0.84 e Å^−^^3^
67 restraints	Absolute structure: Flack (1983), 1930 Friedel pairs
Primary atom site location: structure-invariant direct methods	Flack parameter: 0.05 (3)

### Special details

**Table:**

Geometry. All e.s.d.'s (except the e.s.d. in the dihedral angle between two l.s. planes) are estimated using the full covariance matrix. The cell e.s.d.'s are taken into account individually in the estimation of e.s.d.'s in distances, angles and torsion angles; correlations between e.s.d.'s in cell parameters are only used when they are defined by crystal symmetry. An approximate (isotropic) treatment of cell e.s.d.'s is used for estimating e.s.d.'s involving l.s. planes.
Refinement. Refinement of *F*^2^ against ALL reflections. The weighted *R*-factor *wR* and goodness of fit *S* are based on *F*^2^, conventional *R*-factors *R* are based on *F*, with *F* set to zero for negative *F*^2^. The threshold expression of *F*^2^ > σ(*F*^2^) is used only for calculating *R*-factors(gt) *etc*. and is not relevant to the choice of reflections for refinement. *R*-factors based on *F*^2^ are statistically about twice as large as those based on *F*, and *R*- factors based on ALL data will be even larger.

### Fractional atomic coordinates and isotropic or equivalent isotropic displacement parameters (Å^2^)

**Table:**

	*x*	*y*	*z*	*U*_iso_*/*U*_eq_	Occ. (<1)
Br1	0.70576 (5)	0.22447 (6)	0.7036 (4)	0.0927 (5)	
C1	0.5691 (4)	0.0248 (5)	0.2951 (17)	0.080 (2)	
H1A	0.5861	0.0122	0.1559	0.119*	
H1B	0.5318	0.0605	0.2802	0.119*	
H1C	0.5596	−0.0322	0.3659	0.119*	
C2	0.5941 (4)	0.0721 (5)	0.6628 (15)	0.078 (2)	
H2A	0.5854	0.0088	0.6960	0.116*	
H2B	0.5568	0.1078	0.6790	0.116*	
H2C	0.6255	0.0950	0.7583	0.116*	
C3	0.6796 (4)	0.0348 (5)	0.386 (2)	0.090 (3)	
H3A	0.7120	0.0690	0.4560	0.134*	
H3B	0.6872	0.0342	0.2340	0.134*	
H3C	0.6793	−0.0273	0.4385	0.134*	
C4	0.6175 (3)	0.0794 (4)	0.4297 (12)	0.0541 (16)	
C5	0.6193 (3)	0.1826 (4)	0.3626 (10)	0.0430 (13)	
C6	0.6535 (3)	0.2502 (4)	0.4644 (11)	0.0481 (15)	
C7	0.6560 (3)	0.3424 (4)	0.3944 (11)	0.0445 (13)	
C8	0.5863 (3)	0.2102 (4)	0.1850 (14)	0.0542 (16)	
H8	0.5632	0.1657	0.1131	0.065*	
C9	0.5847 (3)	0.2997 (4)	0.1046 (11)	0.0470 (14)	
C10	0.6206 (2)	0.3676 (4)	0.2096 (10)	0.0412 (12)	
C11	0.6917 (3)	0.4127 (5)	0.4975 (12)	0.0518 (16)	
H11	0.7152	0.3976	0.6177	0.062*	
C12	0.6924 (3)	0.5025 (5)	0.4239 (12)	0.0534 (16)	
H12	0.7152	0.5471	0.4967	0.064*	
C13	0.6581 (3)	0.5270 (4)	0.2360 (11)	0.0444 (13)	
C14	0.6213 (3)	0.4595 (4)	0.1356 (9)	0.0412 (12)	
C15	0.5488 (4)	0.3282 (5)	−0.0776 (13)	0.070 (2)	
H15	0.5243	0.2850	−0.1480	0.084*	
C16	0.5495 (4)	0.4147 (5)	−0.1491 (14)	0.068 (2)	
H16	0.5260	0.4293	−0.2696	0.081*	
C17	0.5850 (3)	0.4861 (5)	−0.0475 (11)	0.0513 (15)	
C18	0.5874 (3)	0.5748 (4)	−0.1191 (11)	0.0519 (15)	
H18	0.5638	0.5904	−0.2386	0.062*	
C19	0.6590 (3)	0.6164 (4)	0.1567 (11)	0.0503 (14)	
H19	0.6840	0.6599	0.2244	0.060*	
C20	0.6236 (3)	0.6438 (4)	−0.0222 (11)	0.0502 (15)	
C21	0.6244 (3)	0.7430 (5)	−0.1038 (9)	0.0588 (18)	
C22	0.6781 (6)	0.7999 (9)	−0.009 (3)	0.067 (5)	0.59 (3)
H22A	0.6705	0.8107	0.1400	0.100*	0.59 (3)
H22B	0.6809	0.8577	−0.0832	0.100*	0.59 (3)
H22C	0.7165	0.7667	−0.0261	0.100*	0.59 (3)
C23	0.5639 (5)	0.7879 (9)	−0.031 (3)	0.064 (4)	0.59 (3)
H23A	0.5298	0.7466	−0.0558	0.096*	0.59 (3)
H23B	0.5573	0.8438	−0.1096	0.096*	0.59 (3)
H23C	0.5664	0.8017	0.1194	0.096*	0.59 (3)
C24	0.6287 (9)	0.7500 (11)	−0.3457 (14)	0.073 (5)	0.59 (3)
H24A	0.6656	0.7191	−0.3942	0.109*	0.59 (3)
H24B	0.6305	0.8137	−0.3868	0.109*	0.59 (3)
H24C	0.5927	0.7218	−0.4092	0.109*	0.59 (3)
C22'	0.6879 (9)	0.790 (3)	−0.103 (6)	0.20 (3)	0.41 (3)
H22D	0.7175	0.7511	−0.0305	0.299*	0.41 (3)
H22E	0.6850	0.8477	−0.0300	0.299*	0.41 (3)
H22F	0.7014	0.7997	−0.2477	0.299*	0.41 (3)
C23'	0.5813 (12)	0.7978 (15)	0.043 (3)	0.070 (7)	0.41 (3)
H23D	0.5419	0.7669	0.0541	0.106*	0.41 (3)
H23E	0.5751	0.8583	−0.0149	0.106*	0.41 (3)
H23F	0.5998	0.8025	0.1828	0.106*	0.41 (3)
C24'	0.5979 (14)	0.7505 (15)	−0.331 (2)	0.073 (7)	0.41 (3)
H24D	0.6186	0.7072	−0.4224	0.110*	0.41 (3)
H24E	0.6044	0.8118	−0.3841	0.110*	0.41 (3)
H24F	0.5541	0.7374	−0.3280	0.110*	0.41 (3)

### Atomic displacement parameters (Å^2^)

**Table:**

	*U*^11^	*U*^22^	*U*^33^	*U*^12^	*U*^13^	*U*^23^
Br1	0.1018 (8)	0.0731 (6)	0.1033 (8)	−0.0084 (4)	−0.0434 (7)	0.0225 (5)
C1	0.100 (6)	0.045 (4)	0.094 (6)	−0.017 (4)	−0.011 (5)	−0.001 (4)
C2	0.104 (6)	0.059 (4)	0.070 (5)	−0.016 (4)	0.009 (5)	0.023 (4)
C3	0.070 (5)	0.044 (4)	0.155 (10)	0.013 (4)	0.019 (6)	0.014 (5)
C4	0.062 (4)	0.038 (3)	0.062 (4)	−0.003 (3)	0.006 (3)	0.007 (3)
C5	0.047 (3)	0.043 (3)	0.039 (3)	−0.005 (2)	0.002 (3)	0.005 (2)
C6	0.052 (3)	0.042 (3)	0.050 (4)	0.006 (3)	−0.012 (3)	0.006 (3)
C7	0.045 (3)	0.039 (3)	0.049 (3)	0.006 (2)	−0.002 (3)	0.007 (3)
C8	0.070 (4)	0.039 (3)	0.054 (4)	−0.008 (3)	0.009 (4)	0.005 (3)
C9	0.054 (3)	0.042 (3)	0.045 (3)	0.000 (3)	−0.009 (3)	−0.002 (3)
C10	0.042 (3)	0.041 (3)	0.041 (3)	0.007 (2)	−0.002 (2)	−0.005 (3)
C11	0.063 (4)	0.042 (3)	0.051 (4)	−0.002 (3)	−0.022 (3)	0.004 (3)
C12	0.065 (4)	0.043 (3)	0.053 (4)	0.001 (3)	−0.013 (3)	−0.003 (3)
C13	0.045 (3)	0.039 (3)	0.049 (3)	0.007 (2)	−0.002 (3)	−0.005 (3)
C14	0.046 (3)	0.043 (3)	0.035 (3)	0.006 (2)	0.004 (2)	0.000 (2)
C15	0.100 (6)	0.061 (4)	0.050 (4)	−0.018 (4)	−0.027 (4)	−0.002 (4)
C16	0.081 (5)	0.059 (4)	0.062 (4)	−0.008 (4)	−0.032 (4)	0.004 (3)
C17	0.054 (4)	0.056 (4)	0.045 (3)	0.004 (3)	−0.006 (3)	0.002 (3)
C18	0.056 (4)	0.050 (4)	0.050 (4)	0.009 (3)	−0.006 (3)	0.010 (3)
C19	0.057 (3)	0.037 (3)	0.057 (4)	0.002 (2)	0.006 (3)	0.001 (3)
C20	0.055 (4)	0.048 (3)	0.048 (4)	0.011 (3)	0.011 (3)	0.003 (3)
C21	0.074 (5)	0.037 (3)	0.065 (5)	0.006 (3)	0.009 (4)	0.002 (3)
C22	0.067 (6)	0.060 (6)	0.073 (6)	−0.006 (4)	−0.002 (4)	0.007 (4)
C23	0.061 (5)	0.061 (5)	0.070 (6)	0.003 (4)	−0.005 (4)	0.003 (4)
C24	0.079 (6)	0.069 (6)	0.071 (6)	−0.007 (4)	0.009 (4)	0.002 (4)
C22'	0.20 (3)	0.20 (3)	0.20 (3)	0.000 (5)	0.000 (5)	0.002 (5)
C23'	0.074 (8)	0.068 (8)	0.070 (8)	0.000 (5)	−0.001 (5)	0.000 (5)
C24'	0.077 (8)	0.071 (7)	0.071 (8)	−0.001 (5)	0.004 (5)	0.008 (5)

### Geometric parameters (Å, °)

**Table:**

Br1—C6	1.905 (6)	C16—C17	1.435 (10)
C1—C4	1.553 (11)	C16—H16	0.9300
C1—H1A	0.9600	C17—C18	1.363 (9)
C1—H1B	0.9600	C18—C20	1.405 (10)
C1—H1C	0.9600	C18—H18	0.9300
C2—C4	1.543 (12)	C19—C20	1.409 (10)
C2—H2A	0.9600	C19—H19	0.9300
C2—H2B	0.9600	C20—C21	1.529 (9)
C2—H2C	0.9600	C21—C24	1.516 (9)
C3—C4	1.509 (10)	C21—C23	1.524 (8)
C3—H3A	0.9600	C21—C22'	1.524 (9)
C3—H3B	0.9600	C21—C23'	1.526 (9)
C3—H3C	0.9600	C21—C24'	1.530 (9)
C4—C5	1.556 (9)	C21—C22	1.536 (8)
C5—C8	1.376 (10)	C22—H22A	0.9600
C5—C6	1.382 (9)	C22—H22B	0.9600
C6—C7	1.409 (8)	C22—H22C	0.9600
C7—C11	1.430 (9)	C23—H23A	0.9600
C7—C10	1.430 (9)	C23—H23B	0.9600
C8—C9	1.394 (9)	C23—H23C	0.9600
C8—H8	0.9300	C24—H24A	0.9600
C9—C10	1.412 (8)	C24—H24B	0.9600
C9—C15	1.436 (10)	C24—H24C	0.9600
C10—C14	1.413 (8)	C22'—H22D	0.9600
C11—C12	1.383 (10)	C22'—H22E	0.9600
C11—H11	0.9300	C22'—H22F	0.9600
C12—C13	1.431 (10)	C23'—H23D	0.9600
C12—H12	0.9300	C23'—H23E	0.9600
C13—C19	1.390 (8)	C23'—H23F	0.9600
C13—C14	1.407 (8)	C24'—H24D	0.9600
C14—C17	1.437 (9)	C24'—H24E	0.9600
C15—C16	1.333 (11)	C24'—H24F	0.9600
C15—H15	0.9300		
			
C4—C1—H1A	109.5	C18—C17—C16	123.9 (6)
C4—C1—H1B	109.5	C18—C17—C14	119.6 (6)
H1A—C1—H1B	109.5	C16—C17—C14	116.4 (6)
C4—C1—H1C	109.5	C17—C18—C20	123.6 (6)
H1A—C1—H1C	109.5	C17—C18—H18	118.2
H1B—C1—H1C	109.5	C20—C18—H18	118.2
C4—C2—H2A	109.5	C13—C19—C20	122.5 (6)
C4—C2—H2B	109.5	C13—C19—H19	118.7
H2A—C2—H2B	109.5	C20—C19—H19	118.7
C4—C2—H2C	109.5	C18—C20—C19	116.1 (6)
H2A—C2—H2C	109.5	C18—C20—C21	122.3 (6)
H2B—C2—H2C	109.5	C19—C20—C21	121.6 (6)
C4—C3—H3A	109.5	C24—C21—C23	108.8 (6)
C4—C3—H3B	109.5	C24—C21—C22'	85.3 (11)
H3A—C3—H3B	109.5	C23—C21—C22'	124.9 (16)
C4—C3—H3C	109.5	C24—C21—C23'	126.9 (11)
H3A—C3—H3C	109.5	C23—C21—C23'	23.1 (9)
H3B—C3—H3C	109.5	C22'—C21—C23'	108.0 (8)
C3—C4—C2	115.5 (8)	C24—C21—C20	113.3 (8)
C3—C4—C1	105.8 (7)	C23—C21—C20	107.2 (7)
C2—C4—C1	104.9 (7)	C22'—C21—C20	115.3 (16)
C3—C4—C5	110.0 (6)	C23'—C21—C20	106.5 (10)
C2—C4—C5	109.2 (6)	C24—C21—C24'	25.3 (8)
C1—C4—C5	111.3 (6)	C23—C21—C24'	86.0 (9)
C8—C5—C6	116.0 (5)	C22'—C21—C24'	107.7 (8)
C8—C5—C4	119.0 (5)	C23'—C21—C24'	107.1 (7)
C6—C5—C4	125.0 (6)	C20—C21—C24'	111.8 (10)
C5—C6—C7	123.6 (6)	C24—C21—C22	107.4 (6)
C5—C6—Br1	122.3 (5)	C23—C21—C22	107.1 (6)
C7—C6—Br1	114.0 (5)	C22'—C21—C22	24.1 (11)
C6—C7—C11	124.0 (6)	C23'—C21—C22	86.8 (11)
C6—C7—C10	118.2 (5)	C20—C21—C22	112.8 (7)
C11—C7—C10	117.8 (5)	C24'—C21—C22	126.5 (11)
C5—C8—C9	125.0 (6)	C21—C22—H22A	109.5
C5—C8—H8	117.5	C21—C22—H22B	109.5
C9—C8—H8	117.5	C21—C22—H22C	109.5
C8—C9—C10	118.1 (6)	C21—C23—H23A	109.5
C8—C9—C15	124.6 (6)	C21—C23—H23B	109.5
C10—C9—C15	117.3 (6)	C21—C23—H23C	109.5
C9—C10—C14	120.9 (5)	C21—C24—H24A	109.5
C9—C10—C7	119.1 (5)	C21—C24—H24B	109.5
C14—C10—C7	120.0 (5)	C21—C24—H24C	109.5
C12—C11—C7	122.0 (6)	C21—C22'—H22D	109.5
C12—C11—H11	119.0	C21—C22'—H22E	109.5
C7—C11—H11	119.0	H22D—C22'—H22E	109.5
C11—C12—C13	120.1 (6)	C21—C22'—H22F	109.5
C11—C12—H12	119.9	H22D—C22'—H22F	109.5
C13—C12—H12	119.9	H22E—C22'—H22F	109.5
C19—C13—C14	120.1 (6)	C21—C23'—H23D	109.5
C19—C13—C12	121.1 (6)	C21—C23'—H23E	109.5
C14—C13—C12	118.8 (5)	H23D—C23'—H23E	109.5
C13—C14—C10	121.2 (5)	C21—C23'—H23F	109.5
C13—C14—C17	118.1 (5)	H23D—C23'—H23F	109.5
C10—C14—C17	120.6 (5)	H23E—C23'—H23F	109.5
C16—C15—C9	122.1 (7)	C21—C24'—H24D	109.5
C16—C15—H15	118.9	C21—C24'—H24E	109.5
C9—C15—H15	118.9	H24D—C24'—H24E	109.5
C15—C16—C17	122.6 (7)	C21—C24'—H24F	109.5
C15—C16—H16	118.7	H24D—C24'—H24F	109.5
C17—C16—H16	118.7	H24E—C24'—H24F	109.5
